# Djulis (*Chenopodium Formosanum*) Prevents Colon Carcinogenesis via Regulating Antioxidative and Apoptotic Pathways in Rats

**DOI:** 10.3390/nu11092168

**Published:** 2019-09-10

**Authors:** Chih-Wei Lee, Hong-Jhang Chen, Gui-Ru Xie, Chun-Kuang Shih

**Affiliations:** 1 School of Nutrition and Health Sciences, College of Nutrition, Taipei Medical University, Taipei 11031, Taiwan; 2 Institute of Food Science and Technology, National Taiwan University, Taipei 10617, Taiwan; 3 School of Food Safety, College of Nutrition, Taipei Medical University, Taipei 11031, Taiwan; 4 Master Program in Food Safety, College of Nutrition, Taipei Medical University, Taipei 11031, Taiwan

**Keywords:** djulis, colon cancer, preneoplastic lesions, chemoprevention, antioxidant, apoptosis

## Abstract

Djulis is a cereal crop rich in polyphenols and dietary fiber that may have nutraceutical activity to prevent colon cancer. This study was designed to examine the preventive effect of djulis on colon carcinogenesis in rats treated with 1,2-dimethylhydrazine (DMH). Rats were fed different AIN-93G-based diets: groups N and DMH were fed AIN-93G diet and groups LD, MD, and HD were fed AIN-93G diet containing 5, 10, and 20% djulis, respectively. All rats except for group N were injected with DMH to induce colon carcinogenesis. After 10 weeks, rats were sacrificed and colon and liver tissues were collected for analysis. The results showed that djulis-treated rats had significantly lower numbers of colonic preneoplastic lesions, aberrant crypt foci (ACF), sialomucin-producing (SIM)-ACF, and mucin-depleted foci. Djulis treatment increased superoxide dismutase and catalase activities in colon and liver. Djulis also reduced p53, Bcl-2, and proliferating cell nuclear antigen expressions and increased Bax and caspase-9 expressions. Besides, phenolic compounds and flavonoids were found rich in djulis. These results demonstrate the chemopreventive effect of djulis on carcinogen-induced colon carcinogenesis via regulating antioxidative and apoptotic pathways in rats. Djulis may have the potential to be developed as a valuable cereal product for chemoprevention of colon cancer.

## 1. Introduction

Colorectal cancer is the third most common cancer worldwide [1]. Many studies have proven that dietary patterns are important progenitors of colon carcinogenesis. It is known that the risk of colon cancer increases with high intake of red meat and animal fat [2]. However, whole grains, vegetables, and fruits reduce the risk of colon cancer [3]. Besides, phenolic compounds and flavonoids in diet also have preventive effect on colon cancer [4,5]. These demonstrated that dietary factors play an important role in progress of colon cancer.

Aberrant crypt foci (ACF), the earliest identifiable preneoplastic lesion in the colon, were shown to be correlated with the development of colon cancer and used as a biomarker to study the effects of dietary factors on colon carcinogenesis in rats [6,7]. Mucin produced by ACF is also affected during the progression of colon cancer [8]. Sialomucin-producing (SIM)-ACF show higher cell proliferation and dysplasia degree than do sulfomucin-producing (SUM)-ACF [9]. Moreover, mucin-depleted foci (MDF), that exhibit the absence or scant production of mucin, carry alterations in cell signaling pathways, and gene mutations, with a frequency similar to that observed in tumors [10].

Excessive levels of reactive oxygen species (ROS) result in high oxidative stress. Oxidative stress alters expression of the p53 protein, brings about increased expression of the antiapoptotic protein Bcl-2 and reduced expressions of the proapoptotic proteins Bax and caspase-9, and leads to colon carcinogenesis [11,12]. It is known that increasing the activities of antioxidant enzymes can reduce the colon cancer risk, as they can protect tissues against ROS damage [13].

Taiwanese chenopod, *Chenopodium formosanum* (djulis), a cereal crop native to Taiwan, has been used as a traditional food by Taiwanese aboriginals for hundreds of years. Djulis have many benefits to health, such as anti-adipogenesis [14] and recovering liver injury [15,16]. Besides this, many studies have found that djulis is rich in betalain which imparts an excellent antioxidative capacity to djulis [17]. Polyphenols, such as rutin and chlorogenic acid, are also found in djulis [18]. Many studies proved that rutin and chlorogenic acid have antitumor properties [19,20]. Therefore, djulis may have the potential for a preventive effect against colon cancer. However, there were no studies showing the preventive effect of djulis on cancer. 

In this study, we employed a rat model with colonic preneoplastic lesions to determine whether djulis can serve as a chemopreventive agent to prevent colon carcinogenesis via protecting rats against oxidative stress and modulating cell proliferation and apoptosis.

## 2. Materials and Methods 

### 2.1. Materials

Djulis was obtained from Sinfong Agritech Co. (Taipei, Taiwan). Iron (III) chloride hexahydrate, methylene blue, and acetic acid were purchased from Nacalai Tesque (Tokyo, Japan), Showa Chemicals (Tokyo, Japan), and Shimakyu Pure Chemicals (Osaka, Japan), respectively. The Bax primary antibody and Bcl-2 primary antibody were purchased from Cell Signaling Technology (Beverly, MA, USA). Glyceraldehyde-3-phosphate dehydrogenase and the caspase-9 primary antibody were purchased from GeneTex (Irvine, CA, USA). The proliferating cell nuclear antigen (PCNA) primary antibody, goat anti-rabbit immunoglobulin G (IgG) secondary antibody, and peroxidase AffiniPure goat anti-mouse IgG were purchased from Abcam (Cambridge, UK), Southern Biotechnology (Birmingham, AL, USA), and Jackson ImmunoResearch (West Grove, PA, USA), respectively. The β-actin primary antibody, 1,2-dimethylhydrazine (DMH), N,N’-dimethyl-p-phenylenediamine, N,N’-dimethyl-m-phenylenediamine, Alcian blue, and the remaining chemicals were purchased from Sigma Chemical (St. Louis, MO, USA).

### 2.2. Experimental Design

The animal study protocol was approved (LAC-2014-0198) by the Institutional Animal Care and Use Committee of Taipei Medical University. Fifty-seven male F344 rats which aged 4–8 week were from the National Laboratory Animal Center (Taipei, Taiwan). Rats were housed in cages maintained at 21 °C and kept to a 12-h light-dark cycle. Rats had free access to food and water. After 1 week of an adaptation period, animals were randomly divided into five groups (with 9 or 12 rats per group) and fed different diets: groups N and DMH were fed AIN-93G diet and groups LD, MD, and HD were fed AIN-93G diet containing 5, 10, and 20% whole djulis, respectively. The diets were adjusted the content of carbohydrates, fat, protein, and dietary fiber according to the addition of different amounts of djulis, so the levels of macronutrients and dietary fiber of all groups were consistent. After feeding the experimental diet for 1 week, all rats except for those in group N were intraperitoneally injected with 1,2-dimethylhydrazine (DMH) (40 mg/kg body weight) once a week for 4 weeks to induce colon carcinogenesis. All rats were sacrificed after being fed for 10 weeks, and colon and liver tissues were collected.

### 2.3. Aberrant Crypt Foci (ACF) Counts in the Colon

The colon was stained with a 0.2% methylene blue solution and ACF were counted using a method described in our previous study [21]. The total number of ACF and the number of aberrant crypts (ACs) in each focus were counted under a light microscope (Nikon, Tokyo, Japan) at 40× magnification. NIS-Elements microscope imaging software (Nikon, Tokyo, Japan) was used to calculate the area of the colon. Data of ACF and ACs are presented as the number/cm^2^.

### 2.4. Identification of Mucin-Producing Aberrant Crypt Foci (ACF) and Mucin-Depleted Foci (MDF)

The colon samples were stained with high-iron diamine alcian blue (HIDAB) as described in our previous study [21] and observed under a light microscope (Nikon, Tokyo, Japan) at 40× magnification. ACF stained dark brown by HIDAB indicated SUM production, while those stained bright or dark blue indicated SIM production. Samples with >85% SIM-producing cells were defined as SIM-ACF, those with >85% SUM-producing cells were defined as SUM-ACF, and those with not more than 85% SIM-producing or 85% SUM-producing cells were defined as mixed-type (MIX)-ACF. Furthermore, MDF were defined as those with very little or no production of mucins. NIS-Elements microscope imaging software (Nikon, Tokyo, Japan) was used to calculate the area of the colon. Data of mucin-producing ACF and MDF are presented as the number/cm^2^.

### 2.5. Measuring Activities of Antioxidant Enzymes

Colon and liver tissues were homogenized in five volumes of buffer (210 mM mannitol, 1 mM EDTA, 70 mM sucrose, and 50 mM phosphate buffer, at pH 7.4) for measurement of antioxidant enzyme activities. The superoxide dismutase (SOD) activity and catalase (CAT) activity assays were both performed using commercial kits (Superoxide Dismutase Assay Kit and Catalase Assay Kit; Cayman Chemical, Ann Arbor, MI, USA) according to the manufacturer’s protocols. 

### 2.6. Western Blot Analysis

Colon and liver tissues were homogenized in five volumes of modified RIPA buffer (0.5 M Tris-HCl at pH 7.4, 1.5 M NaCl, 2.5% deoxycholic acid, 10% NP-40, and 10 mM EDTA) and 10% protease inhibitor cocktail. Homogenates were centrifuged at 10^4^ × g for 15 min at 4 °C, and the supernatants were collected. Bradford protein assay was used to determine the total protein concentration. Protein samples were separated on 10% sodium dodecylsulfate polyacrylamide gels and transferred to polyvinylidene difluoride membranes. Membranes were blocked with 5% bovine serum albumin in Tris-buffered saline containing 1% Tween 20 (TBST) and were incubated overnight at 4 °C with appropriate antibodies for 16 h. After incubation, membranes were washed with TBST and incubated with anti-mouse or anti-rabbit horseradish peroxidase-conjugated secondary antibodies for 1 h, and then washed with TBST. After that, chemiluminescence reagents and chemiluminometer (BioSpectrum AC Imaging System, Ultra-Violet Products, Upland, CA, USA) were used to visualize and detect the immunocomplexes.

### 2.7. Djulis Extraction

The dried whole djulis, djulis husk, and djulis without husk (2 g) were separately soaked with 30 mL of 0.1% HCl/70% methanol. The mixture was extracted by shaker at 70 rpm for 60 min, centrifuged at 2250 × *g* for 10 min and then filtered through Whatman No. 1 filter paper. The filter residue was re-extracted 2 times under the same condition. The combined extracts were evaporated under reduced pressure in a rotary vacuum evaporator. The dry extract was mixed with 3 mL distilled water which was adjusted to pH 2.0 with HC1 to obtain crude extract. The extract was successively partitioned with 6 mL diethyl ether/ethyl acetate (1:1, v/v) for 3 times. The organic layer was collected, concentrated under reduced pressure, and then dissolved in 70% methanol to obtain free phenolics (FP).

For water layer, 0.75 mL of 10 N NaOH was added to make final concentration at 2 N NaOH, and then stirred at room temperature for 24 h, after that, adjusted to pH 2.0 with 6 N HCl and added twice water layer volume of the ether/ethyl acetate (1:1, v/v). The successive partition was repeated 3 times. The organic layer was collected, concentrated under reduced pressure with a reduced pressure concentrator, and then dissolved in 70% methanol to be 10000 μg/mL, which was conjugated base-hydrolyzate (CBH). To obtain conjugated acid-hydrolyzate (CAH), the 6 N HCl was further added to the aqueous layer to a final concentration of 2 N HCl and hydrolyzed in a water bath at 85 °C for 1 h, then adjusted to pH 2 with 10 N NaOH, and added twice water layer volume of the diethyl ether/ethyl acetate (1:1, v/v). The successive partition was repeated for three times. The organic layer was collected, concentrated under reduced pressure in a vacuum concentrator, and then dissolved in 70% methanol to afford 10000 μg/mL.

After extracted with 0.1% HCl/70% methanol, the filter residue was dried in an oven at 35 °C, and then added with 25 mL of 2 N NaOH, and shaken at room temperature for 4 h. The gelatinized sample was added with 100 mL of 70% methanol, stirred with glass rod, and then the supernatant was filtered through Whatman No. 1 filter paper. The supernatant was collected and concentrated under reduced pressure to remove methanol, and the aqueous layer was adjusted to pH 2 with 6 N HCl, added twice water layer volume of the diethyl ether/ethyl acetate (1:1, v/v). The successive partition was repeated for three times. The organic layer was collected, concentrated under reduced pressure in a vacuum concentrator, and then dissolved in 70% methanol to afford 10000 μg/mL, which was bound base-hydrolyzate (BBH). The filter residue was further added with 25 mL of 2 N HCl and hydrolyzed in water bath at 85 °C for 1 h, and then adjusted to pH 2 with 10 N NaOH, and added twice water layer volume of diethyl ether/ethyl acetate (1:1, v/v) The successive partition was repeated for three times. The organic layer was collected, concentrated under reduced pressure on a vacuum concentrator, and then dissolved in 70% methanol to afford 10000 μg/mL, which was bound acid-hydrolyzate (BAH).

### 2.8. Determination of Total Phenolics Content in Djulis Samples

The total phenolic content was determined according to the Folin–Ciocalteu method [22]. Briefly, 50 μL of each sample was separately mixed with 50 μL of Folin–Ciocalteu reagent, and 400 μL of 5% sodium carbonate. After incubation in the dark for 30 min, the absorbance was measured at 750 nm with a microplate reader (Molecular Devices, Sunnyvale, CA, USA). The amount of Folin–Ciocalteu reagent was substituted by the same amount of distilled water in the blank. Gallic acid (250, 200, 150, 100, 50, and 25 μg/mL) was used to make a calibration curve. The total phenolic content was expressed as milligram gallic acid equivalents (GAE) per gram of extract.

### 2.9. Determination of Total Flavonoids Content in Djulis Samples

To determine the total flavonoid content in djulis extract, AlCl_3_ method was used [22]. Briefly, 200 μL of each sample was separately mixed with 500 μL of 5% aluminum chloride, 250 μL of 1 N sodium hydroxide. After incubation for 15 min, the absorbance was measured at 415 nm with a microplate reader (Molecular Devices). The amount of 10% aluminum chloride was substituted by the same amount of distilled water in the blank. Quercetin (250, 200, 150, 100, 50, 25, and 10 μg/mL) was used to make a calibration curve. The total flavonoid content was expressed as milligram quercetin equivalents (QE) per gram of extract.

### 2.10. Statistical Analysis

Experimental data are presented as the mean ± standard deviation (SD). Statistical analyses were performed with SAS software (SAS Institute, Cary, NC, USA). Differences among experimental data were assessed by a one-way analysis of variance (ANOVA) followed by Duncan’s multiple-range test. All p values of <0.05 were considered significant. 

## 3. Results

### 3.1. Numbers of Colonic Aberrant Crypt Foci (ACF) and ACs

**Figure 1:**
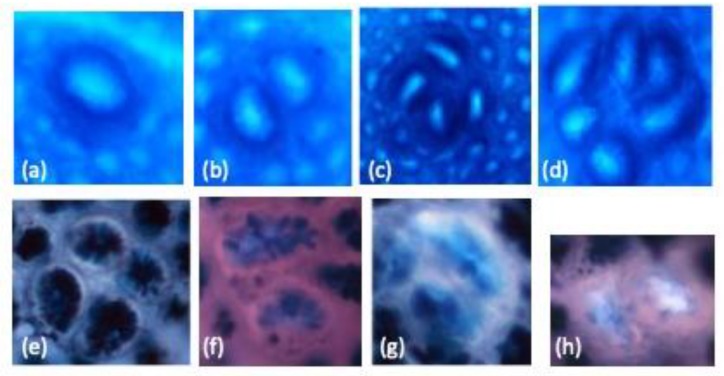
The representatives of aberrant crypt foci (ACF) with one AC (**a**), ACF with two AC (**b**), ACF with four AC (**c**), ACF with six AC (**d**), sulfomucin-producing ACF (SUM-ACF) (**e**), ACF with not more than 85% SIM-producing or 85% SUM-producing cells were defined as mixed-type (MIX)-ACF (**f**), sialomucin-producing ACF (SIM-ACF) (**g**) and mucin-depleted foci (MDF) (**h**) in 1,2-dimethylhydrazine (DMH)-induced colon carcinogenesis in male F344 rats. The original magnification is 100 ×.

There were no significant differences in the feed efficiency among groups (data not shown). The representatives of the ACF, SUM-ACF, MIX-ACF, SIM-ACF, and MDF for this study are shown in Figure 1. As shown in Table 1, the incidence of ACF in all DMH-treated groups was 100% and there was no ACF found in group N. Results showed that groups LD, MD, and HD had significantly lower numbers of colonic ACF and ACs than did group DMH. The inhibitory effect of djulis on ACF was dose dependent. ACF were further distinguished into either small or large ACF according to previous studies [23]. The djulis-treated groups had significantly reduced numbers of one-, two-, three-crypt ACF and also small ACF compared to group DMH. In addition, group HD had a significantly lower number of one-crypt ACF than did group LD (Table 2).

### 3.2. Mucin Production by Aberrant Crypt Foci (ACF) and Mucin-Depleted Foci (MDF)

HIDAB-staining results showed that groups MD and HD had significantly reduced numbers of colonic SUM-ACF compared to group DMH. All djulis-treated groups had significantly lower numbers of colonic MIX-ACF, SIM-ACF, and MDF than did group DMH (Table 3).

DMH treatment significantly reduced activities of both SOD and CAT in the liver and colon of rats in group DMH compared to group N. Group LD exhibited significantly increased CAT activity in colonic tissues compared to group DMH. Moreover, groups MD and HD had significantly increased SOD and CAT activities in the liver and colon compared to group DMH (Table 4).

### 3.4. Expression of Proliferation- and Apoptosis-Related Proteins

**Figure 2:**
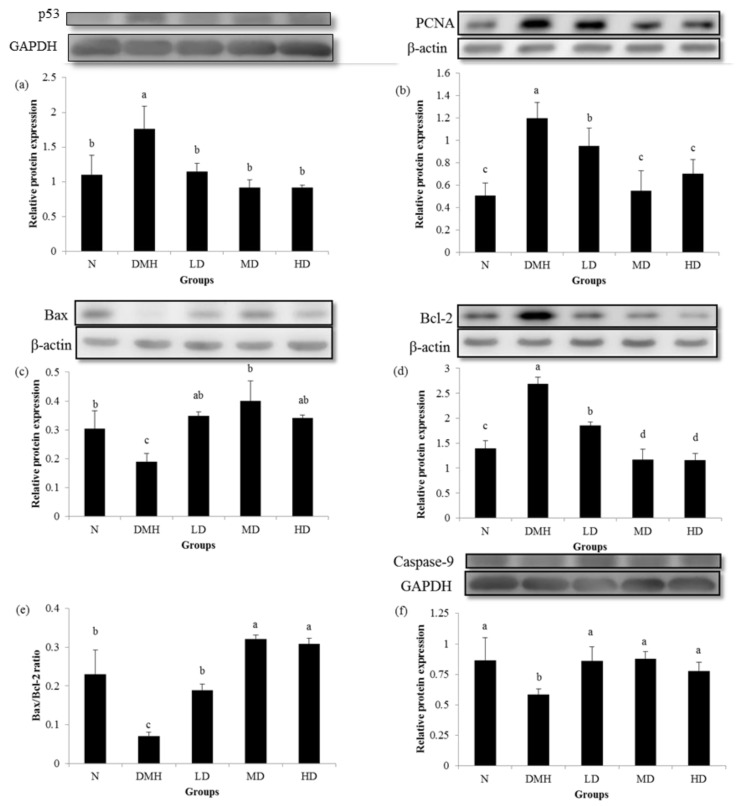
Effects of djulis on p53 (**a**), proliferating cell nuclear antigen (PCNA) (**b**), Bax (**c**), Bcl-2 (**d**), Bax/Bcl-2 (**e**), and caspase-9 (**f**) expressions of the distal colon mucosa of male F344 rats. The bar represents the mean ± SD (*n* = 3–5). Bars with the different letter are significantly different from one another as determined by a one-way ANOVA followed by Duncan’s multiple-range test, *p* < 0.05. All rats except those in group N were administered 1,2-dimethyl hydrazine (DMH). N, AIN-93G diet; DMH, AIN-93G diet; LD, AIN-93G diet containing 5% djulis; MD, AIN-93G diet containing 10% djulis; HD, AIN-93G diet containing 20% djulis.

Figure 2. showed that expression of the p53, antiapoptosis-related Bcl-2 and proliferation-related PCNA proteins were significantly higher in group DMH compared to group N, whereas groups LD, MD and HD had significantly reduced expressions of the p53, Bcl-2 and PCNA proteins compared to group DMH. Groups LD, MD, and HD had significantly increased expressions of the proapoptosis-related Bax, caspase-9 proteins, and Bax/Bcl-2 ratio, which were reduced by DMH administration in group DMH.

### 3.5. Total Phenolics Content and Total Flavonoids Content in Djulis

Table 5 and Table 6 showed that total phenolics and total flavonoids are rich in the husk of djulis. In addition, whole djulis has more total phenolics content than djulis without husk, and there is a similar result in total flavonoids content in djulis samples.

## 4. Discussion

This study showed that djulis inhibited the formation of colonic preneoplastic lesions (ACF, Acs, and MDF) in DMH-induced colon carcinogenesis in rats. Simultaneously, djulis increased the activities of antioxidant enzymes (CAT and SOD) and the expressions of proapoptosis-related proteins (Bax and caspase-9) in the colon of rats. Djulis also inhibited the expressions of p53, a proliferation-related protein (PCNA), and an antiapoptosis-related protein (Bcl-2).

A previous study showed that it took about 30 days to form ACF in a rodent model of chemically induced carcinogenesis [24]. In this study, we used DHM to establish a colon carcinogenesis rat model and the incidences of ACF were 100% in all DMH-treated groups, which illustrates that the present study used a stable colon carcinogenesis model.

It was proven that increasing numbers of ACF appearing in the colon results in an increased risk of colon cancer [25]. The present study showed that all djulis-treated groups had significant reductions in the total number of ACF, and the high-dose djulis-treated group showed much-stronger inhibitory effects than did the low-dose djulis-treated group. In the beginning of colon carcinogenesis, ACF appear as a single crypt and then become ACF with more crypts via a ‘mechanism of crypt fission’. As in more-advanced stages of colon cancer, ACF grow with more crypts [26]; besides, greater numbers of large ACF (with more than four crypts) lead to higher risk of colon tumor formation [27]. The present study found that djulis reduced the total number of ACF and also produced a significant reduction in the number of small ACF. Although djulis showed no effect on large ACF, all djulis-treated groups showed inhibitory effects on the formation of small ACF which were predicted to progress to dysplasia; therefore, inhibiting the formation of small ACF may be considered an effective way to prevent colon carcinogenesis [23]. In the present study, djulis reduced the numbers of total ACF, total ACs and small ACF, suggesting that djulis can prevent colon carcinogenesis at an early stage of ACF development.

Changes in the secretion or type of mucin were discovered during the progression of colon cancer and result in abnormal cell signaling or function in colonic mucosa [28]. A previous study showed that compared to normal colonic tissues, human colorectal adenocarcinomas had significant hypersecretion of SIM and reduced SUM [29]. Rat model of colon carcinogenesis also found that a chemical carcinogen reduced the proportion of SUM-ACF and increased the proportion of SIM-ACF; moreover, SIM-ACF had higher risks of developing into colon tumors [8,30]. MDF which are high-grade dysplasia and considered advanced preneoplastic lesions have been widely used as a precursor to evaluate colon cancer risk in many studies [31]. In this study, all djulis-treated groups exhibited significantly reduced numbers of MIX-ACF, SIM-ACF, and MDF, indicating that djulis may protect against colon carcinogenesis via modulating colonic mucin expression.

Studies discovered that activities of antioxidant enzymes were lower in the plasma of colon cancer patients and also were decreased in the liver and colon of DMH-treated rats [32,33]. Therefore, increased activities of antioxidative system, especially SOD and CAT, play an important role in preventing tissues from damage caused by oxidative stress [12,34]. In this study, the number of ACF was negatively correlated with colonic activities of SOD (*r* = −0.72, *p* = 0.0003) and CAT (*r* = −0.71, *p* = 0.00021), while djulis promoted the activities of colonic and hepatic SOD and CAT in rats. The results are consistent with previous studies that chemopreventive agents protect against colon cancer via increasing SOD and CAT activities [13,33]. Our results demonstrated that djulis can defend against oxidative stress induced by DMH in the colon and liver of rats.

Mutations in the p53 tumor suppressor gene are the most common gene mutations in human cancer, and abnormal function of the p53 protein results in it promoting the progression and metastasis of colon cancer instead of suppressing tumor growth [35,36]. Although we did not detect the mutations of p53 gene directly in this study, a previous study suggested that DMH may cause mutations of the p53 gene, as they found that the expression of p53 protein increased in DMH-induced colon carcinogenesis in a rat model [37]. There were similar results in this study: the DMH-treated group exhibited an increase of p53 protein expression compared to the normal group, while all djulis-treated groups exhibited low expression of the p53 protein which did not significantly differ from the normal group. Another study showed that inhibiting the abnormal expression of p53 induced apoptosis and inhibited the growth of human cancer cells [38]. Moreover, expression of the p53 protein was positively correlated with the number of MDF (*r* = 0.60, *p* = 0.0235) and the expression of PCNA protein (*r* = 0.63, *p* = 0.0365). These results suggest that DMH may cause mutations of p53 gene and abnormal expression of p53 protein, and djulis could correct the abnormal expression of p53 protein in the progression of colon cancer.

In this study, DMH significantly induced Bcl-2 expression and reduced Bax and caspase-9 expressions in the colon of rats compared to the normal group. These results were consistent with a previous study of DMH-induced colon carcinogenesis in rats [39]. The Bax/Bcl-2 ratio can also be used to evaluate cell apoptosis, while its elevation induces apoptosis of cancer cells [40]. Regulation of Bax, Bcl-2, and caspase-9 plays an important role in activating the intrinsic apoptosis pathway. A recent study by Shen et al. (2010) showed that downregulation of caspase-9 frequently appeared in patients with colorectal cancer and was correlated with poor clinical outcomes [41]. In this study, djulis treatment inhibited Bcl-2 expression, promoted Bax and caspase-9 expressions, and raised the Bax/Bcl-2 ratio. These results were similar to phenomena reported in other studies using chemopreventive agent-treated models of colon carcinogenesis [42,43]. Overexpression of PCNA was a common event in chemical carcinogen-induced colon carcinogenesis in previous studies [44,45]. In this study, PCNA expression was induced in group DMH, while the djulis-treated groups showed significant reductions in PCNA expression.

To understand bioactive compounds in djulis, we further examined the total phenolics and total flavonoids content in djulis samples. We found that whole djulis are rich in total phenolics and total flavonoids content. Many evidences supported that phenolic compounds could regulate apoptosis and proliferation of colon cancer cells in part through oxidant-mediated mechanisms [5]. Studies also found a positive correlation between a flavonoid-rich diet and lower risk of colon cancer [4]. Therefore, phenolic compounds and flavonoids have an anticancer effect. Taken together, phenolics and flavonoids in djulis may play an important role in preventing colon cancer in this study. However, further studies on bioactive compounds in the extract of djulis samples are still needed, and our research team is working on it.

## 5. Conclusions

In conclusion, these results demonstrate that djulis which is rich in total phenolics and total flavonoids content can protect rats against oxidative stress and regulate antiapoptosis-related, proapoptosis-related and proliferation-related proteins to prevent colon cancer progression. To our knowledge, this is the first study to report the chemopreventive effect of djulis on carcinogen-induced colon carcinogenesis in rats. Therefore, djulis may be a promising valuable cereal product for chemoprevention of colon cancer in the future. Research should further explore the detail mechanisms and effects of djulis on preventing colon cancer in humans.

## Figures and Tables

**Table 1:** Effect of djulis on 1,2-dimethyl hydrazine (DMH)-induced aberrant crypt foci (ACF) and aberrant crypts (ACs) (number/cm^2^) in the colon of male F344 rats ^1.^

Group ^2^	ACF Incidence (% of Animals with ACF)	Number of ACF	Number of ACs	Crypt Multiplicity (Number of ACs/Focus)
DMH	100%	7.8 ± 1.3 ^a^	19.2 ± 3.4 ^a^	2.5 ± 0.3
LD	100%	4.5 ± 1.1 ^b^	10.7 ± 2.4 ^b^	2.4 ± 0.2
MD	100%	4.3 ± 0.7 ^bc^	11.2 ± 2.5 ^b^	2.6 ± 0.2
HD	100%	3.4± 0.8 ^c^	8.7± 2.6 ^b^	2.5 ± 0.2

^1^ All values are the mean ± SD (*n* = 9). Values followed by different superscripts within the same column are significantly different (*p* < 0.05) determined by a one-way ANOVA followed by Duncan’s multiple-range test, *p* < 0.05. ^2^ All rats were administered 1,2-dimethyl hydrazine (DMH). DMH, AIN-93G diet; LD, AIN-93G diet containing 5% djulis; MD, AIN-93G diet containing 10% djulis; HD, AIN-93G diet containing 20% djulis.

**Table 2:** Effect of djulis on 1,2-dimethyl hydrazine (DMH)-induced aberrant crypt foci (ACF) (number/cm^2^) according to various sizes of crypts in the colon of male F344 rats ^1^.

Group ^2^	Number of Focus Containing			Small ACF	Large ACF
	1 crypt	2 crypts	3 crypts	(≤3 crypts)	(≥4 crypts)
DMH	1.3 ± 0.6 ^a^	2.9 ± 0.9 ^a^	2.6 ± 0.7 ^a^	6.7 ± 1.1 ^a^	1.0 ± 0.6
LD	0.9 ± 0.4 ^b^	1.9 ± 0.7 ^b^	1.0 ± 0.3 ^bc^	3.8 ± 1.1 ^b^	0.7 ± 0.3
MD	0.6 ± 0.2 ^bc^	1.5 ± 0.4 ^b^	1.3 ± 0.4 ^b^	3.4 ± 0.5 ^bc^	0.9 ± 0.4
HD	0.4 ± 0.2 ^c^	1.5 ± 0.4 ^b^	0.8 ± 0.3 ^c^	2.7 ± 0.5 ^c^	0.7 ± 0.4

^1^ All values are the mean ± SD (*n* = 9). Values followed by different superscripts within the same column are significantly different (*p* < 0.05) determined by a one-way ANOVA followed by Duncan’s multiple-range test, *p* < 0.05. ^2^ All rats were administered 1,2-dimethyl hydrazine (DMH). DMH, AIN-93G diet; LD, AIN-93G diet containing 5% djulis; MD, AIN-93G diet containing 10% djulis; HD, AIN-93G diet containing 20% djulis.

**Table 3:** Effect of djulis on the number of 1,2-dimethyl hydrazine (DMH)-induced aberrant crypt foci (ACF) (number/cm^2^) according to the type of mucin produced by foci and mucin-depleted foci (MDF) in the distal colon of male F344 rats^1.^

Group ^2^	Number of ACF Producing ^3^			MDF
	SUM	MIX	SIM	
DMH	8.6 ± 1.9 ^a^	1.4 ± 0.9 ^a^	1.8 ± 0.9 ^a^	0.7 ± 0.3 ^a^
LD	6.6 ± 2.9 ^ab^	0.5 ± 0.2 ^b^	0.4 ± 0.3 ^b^	0.1 ± 0.1 ^b^
MD	4.8 ± 1.9 ^b^	0.8 ± 0.3 ^b^	0.5 ± 0.4 ^b^	0.1 ± 0.1 ^b^
HD	5.1 ± 2.3 ^b^	0.7 ± 0.4 ^b^	0.4 ± 0.4 ^b^	0.1 ± 0.1 ^b^

^1^ All values are the mean ± SD (*n* = 9). Values followed by different superscripts within the same column are significantly different (*p* < 0.05) determined by a one-way ANOVA followed by Duncan’s multiple-range test, *p* < 0.05. ^2^ All rats were administered 1,2-dimethyl hydrazine (DMH). DMH, AIN-93G diet; LD, AIN-93G diet containing 5% djulis; MD, AIN-93G diet containing 10% djulis; HD, AIN-93G diet containing 20% djulis. ^3^ SUM, sulfomucin; MIX, mixed sulfomucin and sialomucin; SIM, sialomucin.3.3. CAT and SOD Activities in the Colon and Liver.

**Table 4:** Effects of djulis on activities of catalase (CAT) and superoxide dismutase (SOD) in the colon and liver of male F344 rats^1.^

Group ^2^	Colon		Liver	
	CAT ^3^ (U/mg Protein)	SOD ^4^ (U/mg Protein)	CAT (U/mg Protein)	SOD (U/mg Protein)
N	29.6 ± 7.7 ^c^	4.1 ± 0.7 ^b^	2409 ± 281 ^b^	30.6 ± 2.4 ^a^
DMH	20.8 ± 6.7 ^d^	3.0 ± 0.6 ^c^	1852 ± 251 ^c^	21.1 ± 3.7 ^b^
LD	29.9 ± 4.0 ^c^	3.4 ± 0.3 ^bc^	1649 ± 130 ^c^	20.1 ± 2.3 ^b^
MD	47.5 ± 2.9 ^b^	5.3 ± 0.3 ^a^	2328 ± 366 ^b^	27.9 ± 3.3 ^a^
HD	58.1 ± 6.2 ^a^	5.8 ± 0.9 ^a^	3282 ± 312 ^a^	28.5 ± 4.4 ^a^

^1^ All values are the mean ± SD (*n* = 9). Values followed by different superscripts within the same column are significantly different (*p* < 0.05) determined by a one-way ANOVA followed by Duncan’s multiple-range test, *p* < 0.05. ^2^ All rats were administered 1,2-dimethyl hydrazine (DMH). DMH, AIN-93G diet; LD, AIN-93G diet containing 5% djulis; MD, AIN-93G diet containing 10% djulis; HD, AIN-93G diet containing 20% djulis. ^3^ One unit of SOD is defined as the amount of enzyme needed to exhibit 50% dismutation of the superoxide radical. ^4^ One unit of CAT is defined as the amount of enzyme needed to form 1.0 nmol of formaldehyde per minute at 25 °C.

**Table 5:** Total phenolics content in the 70% methanol extract in djulis samples ^1.^

Sample	Free Form	Conjugated Form		Bound Form		Total Phenolics
		Base-Hydrolyzable	Acid-Hydrolyzable	Base-Hydrolyzable	Acid-Hydrolyzable	
Whole djulis	666.92 ± 17.55	850.39 ± 51.28	1949.53 ± 54.31	1311.87 ± 11.00	438.38 ± 35.03	5217.10
Djulis husk	1823.30 ±25.08	2414.63 ± 62.61	4570.61 ± 77.72	1820.47 ± 20.55	96.40 ± 13.54	10725.40
Djulis without husk	308.61 ± 5.59	353.47 ± 6.25	1159.80 ± 11.90	421.87 ± 31.94	325.84 ± 11.14	2569.59

^1^ Unit: µg gallic acid equivalent/g sample.

**Table 6:** Total flavonoids content in the 70% methanol extract in djulis sample ^1.^

Sample	Free Form	Conjugated Form		Bound Form		Total Flavonoids
		Base-Hydrolyzable	Acid-Hydrolyzable	Base-Hydrolyzable	Acid-Hydrolyzable	
Whole djulis	582.25 ± 42.96	670.96 ± 31.78	750.75 ± 46.16	720.66 ± 27.11	373.88 ± 490.41	3098.80
Djulis husk	1904.03 ± 32.75	1790.28 ± 20.15	2282.50 ± 53.77	996.93 ± 21.82	509.38 ± 689.50	7483.10
Djulis without husk	296.26 ± 5.32	137.23 ± 12.60	363.41 ± 6.66	198.90 ± 14.00	106.45 ± 130.74	1102.24

^1^ Unit: µg quercetin equivalent/g sample.
